# Protective effect of edaravone for tourniquet-induced ischemia-reperfusion injury on skeletal muscle in murine hindlimb

**DOI:** 10.1186/1471-2474-14-113

**Published:** 2013-03-27

**Authors:** Kazuichiro Hori, Masaya Tsujii, Takahiro Iino, Haruhiko Satonaka, Takeshi Uemura, Koji Akeda, Masahiro Hasegawa, Atsumasa Uchida, Akihiro Sudo

**Affiliations:** 1 Department of Orthopaedic Surgery, Graduate School of Medicine Mie University, 2-174 Edobashi, Tsu city, Mie prefecture 514-8507, Japan; 2 Mie University, Mie, Japan

**Keywords:** Ischemia-reperfusion injury, Skeletal muscle, Free radical scavenger, Edaravone, iNOS

## Abstract

**Background:**

Studies have shown that ischemia-reperfusion (I/R) produces free radicals leading to lipid peroxidation and damage to skeletal muscle. The purposes of this study were 1) to assess the histological findings of gastrocnemius muscle (GC) and tibialis anterior muscle (TA) in I/R injury model mice, 2) to histologically analyze whether a single pretreatment of edaravone inhibits I/R injury to skeletal muscle in murine models and 3) to evaluate the effect of oxidative stress on these muscles.

**Methods:**

C57BL6 mice were divided in two groups, with one group receiving 3 mg/kg intraperitoneal injections of edaravone (I/R + Ed group) and the other group receiving an identical amount of saline (I/R group) 30 minutes before ischemia. Edaravone (3-methy-1-pheny1-2-pyrazolin-5-one) is a potent and novel synthetic scavenger of free radicals. This drug inhibits both nonenzymatic lipid peroxidation and the lipoxygenase pathway, in addition to having potent antioxidant effects against ischemia reperfusion. The duration of the ischemia was 1.5 hours, with reperfusion at either 24 or 72 hours (3 days). Specimens of gastrocnemius (GC) and anterior tibialis (TA) were removed for histological evaluation and biochemical analysis.

**Results:**

This model of I/R injury was highly reproducible in histologic muscle damage. In the histologic damage score, the mean muscle fibers and inflammatory cell infiltration in the I/R + Ed group were significantly less than the corresponding values of observed in the I/R group. Thus, pretreatment with edaravone was observed to have a protective effect on muscle damage after a period of I/R in mice. In addition, the mean muscle injury score in the I/R + Ed group was also significantly less than the I/R group. In the I/R + Ed group, the mean malondialdehyde (MDA) level was lower than in the I/R group and western-blotting revealed that edaravone pretreatment decreased the level of inducible nitric oxide synthase (iNOS) expression.

**Conclusions:**

Edaravone was found to have a protective effect against I/R injury by directly inhibiting lipid peroxidation of the myocyte by free radicals in skeletal muscles and may also reduce the secondary edema and inflammatory infiltration incidence of oxidative stress on tissue.

## Background

Ischemia-reperfusion (I/R) injury refers to tissue damage caused when blood returns to a tissue after a period of ischemia [1]. Reperfusion triggers a cascade of acute inflammatory events, leading to cellular death and resulting in tissue dysfunction and necrosis [1]. Most research concerning I/R injury has focused on the heart, kidney, liver, brain, lung and intestines since these tissues are considered vital organs [2-4]. I/R injury to skeletal muscles is also a serious problem however, and can cause local disturbances as well as systemic problems such as crush syndrome due to leakage from muscle cells into circulating blood. I/R injuries of skeletal muscles are commonly seen in a variety of trauma and other injuries including muscle damage, transplantations, plastic surgery and limb surgery with extended tourniquet application [5-7].

Although the pathophysiology of I/R injuries has not been fully elucidated due to complex interactions of inflammatory and immunologic signaling pathways [8], it is clear that accumulated free radicals and anaerobic metabolites during ischemia cause the migration of white blood cells and the release of inflammatory factors such as interleukin and free radicals in the reperfusion phase [9-13]. These compounds eventually damage muscle tissues, resulting in irrecoverable muscle damage and general complications [14-16]. One of the strategies of treatment for I/R injury likely to be considered is scavenging free radicals, because I/R injury can activate pathways generating reactive oxygen species with the ability to break down cell membranes [14,17]. In fact, there have been several reports in which a scavenger of free radicals was effective in reducing I/R injuries. Elmali et al. stated that resveratrol, which was previously shown to have free radical scavenging and antioxidant properties in various tissues, protected the skeletal muscles against I/R injuries [18]. Ozyurt et al. described how caffeic acid phenethyl ester (CAPE) protected skeletal muscles from reperfusion injury, and how this protective effect can probably be ascribed to CAPE’s free radical scavenging activity [19].

Edaravone (3-methy-1-pheny1-2-pyrazolin-5-one) is a potent and novel synthetic scavenger of free radicals. This drug inhibits both nonenzymatic lipid peroxidation and the lipoxygenase pathway, in addition to having potent antioxidant effects against ischemia reperfusion-induced vascular endothelial cell injury, delayed neuronal death, brain edema, and concomitant neurological deficits [20-23]. In Japan, edaravone was approved in 2002 for use in treating acute brain infarctions and has been reported to have anti-stroke neuro-protective effects [20-23]. Therefore, we hypothesized that edaravone could protect skeletal muscles from I/R injury in an *in vivo* model.

The purposes of this study were 1) to assess the histological findings of gastrocnemius muscle (GC) with many slow fibers and tibialis anterior muscles (TA) with many fast fibers in using the Crawford model of I/R injury [5], 2) to histologically analyze whether a single pretreatment of edaravone inhibits I/R injury on skeletal muscle in these mouse models, and 3) to evaluate the effect of oxidative stress on these muscles.

## Methods

### Subjects

Forty C57BL6 mice (8-10 w, male, 22-28 g; SLC, Hamamatsu, Japan) were used in all experiments of this study. The animals were housed in a temperature-controlled environment and maintained on a 12 hour light–dark cycle with food and water available ad libitum. The experimental protocol was approved by the committee of animal research at Mie University.

### Animal models

Animals were deeply anesthetized with an intraperitoneal injection of pentobarbital sodium (0.05 mg/g body weight). Bands were applied to the left proximal thighs of 40 animals by 4.5 oz orthodontic rubber bands (ORB; American Orthodontics, Sheboygan, WI) as described by Crawford et al. [5]. Intraperitoneal injection of edaravone 3 mg/kg (I/R + Ed group) or injection of the same amount of saline (I/R group) was administered 30 minutes before rubber band application. The period of the ischemia was 1.5 hours, and the rubber band was released and specimens were collected with reperfusion at either 24 hours (n=10 in each group) or 72 hours (n=10 in each group). Mice remained anesthetized throughout the duration of ischemia with supplemental anesthesia (pentobarbital sodium) as needed. Then, reperfusion was performed by cutting the band with scissor. Specimens of gastrocnemius (GC), and anterior tibialis (TA) were removed. Under deep anesthesia mice were pinned and using a microscope, muscles were carefully excised. The wet weight of the resected muscles was measured.

### Drug administration methods

Edaravone was provided by Mitsubishi Tanabe Pharma Corporation (Tokyo, Japan). It was dissolved in 1N NaOH that was titrated to pH 7.4 with 1N HCL to prepare a final concentration of 0.3 mg/ml. Finally, edaravone was injected intraperitoneally at 3 mg/kg. Intraperitoneal injection of the same amount of saline (I/R group) was administered. The application of drugs was performed under the deep anesthesia before the ischemia by the tourniquet.

### Histological analysis

The ten GC and TA samples (n=5 in each group 24 h, 72 h) harvested from each group were immediately fixed in 4% paraformaldehyde and stored at 4°C overnight. The specimens were cut transversely at 4 μm on a microtome and stained with hematoxylin and eosin (HE). After dewaxing in xylene and rehydration in graded methanol (99% to 70% (v/v)) followed by distilled water, HE staining was performed. The stained slides were examined at 200× magnification on microscopes equipped with a digital camera (BX50; Olympus, Tokyo, Japan). Using a standardized method, each muscle was divided into 15 fields for GC samples and 10 fields for TA samples that were photographed using a CoolSNAP color camera (Roper Scientific, Tuscan, AZ, USA) and analyzed with the software program RS Image (Roper Scientific).

### Histologic grading

In tissue sections stained with HE, a histological damage score was assigned on the following scales: disorganization and degeneration of the muscle fibers (0: Normal, 1: Mild, 2: Moderate, 3: Severe); Inflammatory cell infiltration (0: Normal, 1: Mild, 2: Moderate, 3: Severe) [24]. To test for reproducibility we reviewed our grading with 2 additional laboratory members unaware of the experimental design.

### Histologic injury severity score for muscles

The absolute injury score of each muscle was determined by a method similar to McCormack et al. [25]. Every myocyte in all 15 (GC) or 10 (TA) photograph fields was scored. The muscle injury score was expressed as a percentage, obtained by the number of injured myocytes divided by the total number of myocytes scored within the all fields. The intraobserver and interobserver reliability correlation coefficients of muscle injuries at 2 times were excellent (K= 0.98, 0.90, respectively), as determined by the Cohen kappa correlation coefficient.

### Measurement of malondialdehyde (MDA)

MDA is one of the end products of lipid peroxidation, and has been widely used to determine lipid peroxidation levels [26,27]. The lipid peroxidation was assessed by measuring the MDA content of tissue using a thiobarbituric acid (TBA) assay kit (Northwest Life Science Specialties LLC, Vancouver, WA, USA). The harvested muscle (n=5 in each group 24 h) were immediately frozen in liquid nitrogen after washing with 0.9% NaCl. These resected muscles were then homogenized using a Cryopress (Microtech, Chiba, Japan)、 and stirred in an assay buffer (phosphate buffer, pH 7.0 with EDTA) for 1 hour. Butanol was then added to the sample to remove hemoglobin, because malondialdehyde (MDA) or MDA-like substances and TBA can react, producing a pink pigment in the TBA test reaction. The precipitate was centrifugally pelleted (3 minutes at 10,000 g), and an aliquot of the supernatant was reacted with an equal volume of TBA at 60°C for 60 min. After cooling, sample absorbance at 540 nm was measured. The results were expressed in nmol /mg protein for tissue samples, using the standard graphic prepared according to the measurements done with a standard solution. (Solutions 0 to 4)

### Western blotting

Inducible nitric oxide synthase (iNOS) is a messenger protein involved in immune response, which produces large amounts of NO [28,29]. Expression of iNOS was analyzed by western blotting. Harvested muscle (n=5 in each group 24 h) were frozen in liquid nitrogen, homogenized using a Cryopress (Microtech, Chiba, Japan) and stirred in the RIPA buffer (10 mMTrisHCl (PH 7.4), 1% NP40, 0.1% Sodium deoxycolate, 0.1% Sodium dodecyl sulfate (SDS), 0.15M NaCl, 1mM EDTA, 10 ug/ml aprotinine) for 1 hour. The supernatants were separated by Sodium dodecyl sulfate Polyacrylamide gel electrophoresis (SDS–PAGE), transferred to nitrocellulose membranes, and immunoblotted with primary antibodies. The primary antibody was polyclonal rabbit antiserum against iNOS (Affinity BioReagents, Golden, CO, USA). The bands were visualized using the ECL Western blotting detection system (GE Healthcare UK Ltd., Buckinghamshire, England) and were detected by an LAS-4000 imager (Fujifilm, Tokyo, Japan).

### Statistical analysis

Stat View 5.0 for Windows (SAS Institute, NC, USA) was used for statistical analysis. Data were analyzed using the Mann–Whitney *U*-test or by calculating Spearman’s coefficient of rank correlation. Values of p<0.05 were deemed statistically significant.

## Results

### The degree of muscle injuries in Crawford models was highly reproducible with few histological differences between subjects

**Figure 1:**
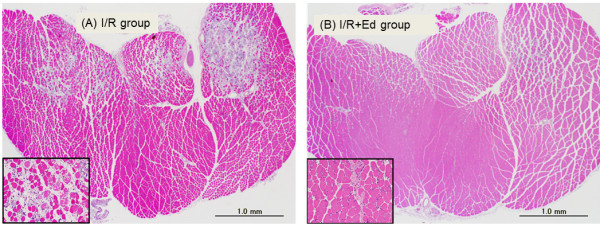
**Histological analysis 72hours (3days) after reperfusion in GC samples. (A)** I/R group: muscle tissue had areas of gross focal and regional fiber necrosis and degeneration, marked cellular infiltration, and loss of tissue structure. **(B)** I/R + Ed group: less muscle damages and uniform distribution comparison to the I/R group.

None of the animals died and none required respiratory support or life support medication during experimental procedures in the 1.5 h model for this study. Histologically, uninjured muscle fibers were characterized as having well defined borders, consistent texture, and uniformity throughout the muscle fiber without holes or breaks. On the other hand, both GC and TA muscles in the I/R group exhibited destruction of muscle fibers with edema and inflammatory cell infiltration 24 hours after reperfusion. In addition, these findings were more pronounced 3 days after reperfusion (Figure 1A,B). Disorganization of muscle fibers and inflammatory cell infiltration was more severe in the center of muscles compared to in the periphery. There was no thrombosis in arteries and also no Deep Venous Thrombosis (DVT) observed. The degree of injuries was largely uniform in most subjects, and all subjects had a high histologic damage score 3 days after I/R injury. This model of I/R injury was highly reproducible in histologic muscle damage just as in the study by Crawford et al. [5].

### Application of edaravone significantly inhibited I/R injury of skeletal muscles in histological analysis

**Figure 2:**
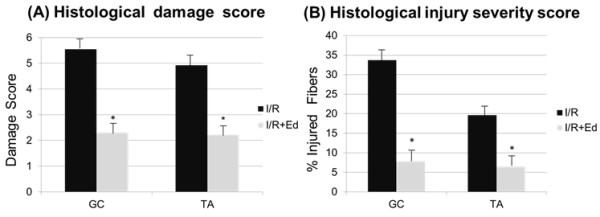
**Histological grading and Histological injury severity score 72hours (3days) after reperfusion.** (**A**) I/R + Ed group histological damage score was significantly less than in the I/R group. (P<0.001) (**B**) Histological injury severity score expressed as percent injured myocytes, showed statistical difference between the I/R and I/R + Ed groups in both GC and TA samples. (P<0.001).

**Figure 3:**
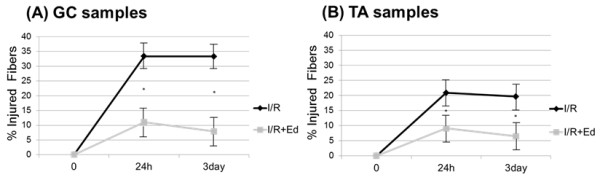
**Histological assessment percentage injury over time.** (**A**) GC samples: The I/R + Ed group showed a significantly a lower injury percentage at 24h, and continuously decreased injury rate at 72h. (P<0.001) (**B**) TA samples: The I/R + Ed group similary showed significantly a lower injury percentage and a decreasing injury rate at the 72h sample time. (P<0.001).

In contrast to the muscle tissues observed after I/R injury in the I/R group (Figure 1A), damage to muscle fibers was rarely observed in the I/R + Ed group. There were however some cases of inflammatory cell infiltration and edema between muscle fibers (Figure 1B). In the histologic damage score, the muscle fibers and inflammatory cell infiltration in the I/R + Ed group were 2.31 ± 0.569 for GC and 2.22 ± 0.465 for TA, both of which were significantly less than the corresponding values of 5.55 ± 0.501 (GC) and 4.92 ± 0.853 (TA) observed in the I/R group. (P<0.001) (Figure 2A) Thus, pretreatment with edaravone was observed to have a protective effect on muscle damage after a period of I/R in mice. In addition, the mean muscle injury score in the I/R + Ed group was 11.1% (116/1051) in GC and 9.1% (83/903) in TA, also significantly less than the 33.4% (363/1085) and 20.9% (193/923) seen in the I/R group. (P<0.001) The difference was also present 3 days after reperfusion, in which application of edaravone inhibited muscle damage from the 33.7% (394/1179) in GC and 19.7% (192/947) in TA seen in the I/R group to 7.9% (93/1178) and 6.5% (62/957). (P<0.001) (Figure 2B) Histological findings indicate that the scavenging of free radicals by edaravone may inhibit inflammatory cascades. (Figure 3A,B)

### The scavenging of free radicals by edaravone not only directly inhibited I/R injury to skeletal muscles but also protected myocytes via indirect transduction of oxidative stress

**Figure 4:**
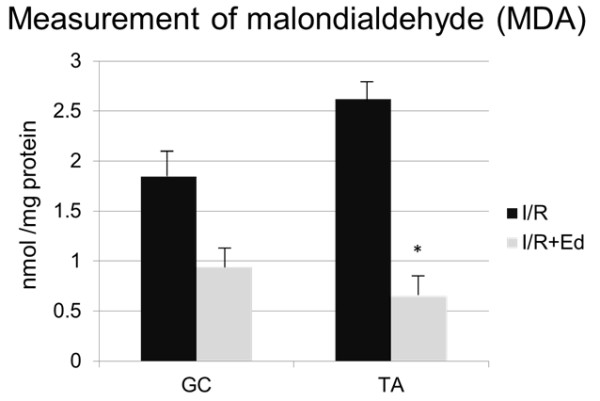
**Malondialdehyde (MDA) concentration in GC and TA 24hours (1day) after reperfusion.** The I/R + Ed group, the mean MDA level was lower than in the I/R group. There was a tendency towards lower peroxidation levels observed in the GC samples of the I/R + Ed group, but it was not statistically significant. (p = 0.08) The difference in the TA peroxidation levels however was significantly lower in the I/R + Ed group. (p<0.05).

**Figure 5:**
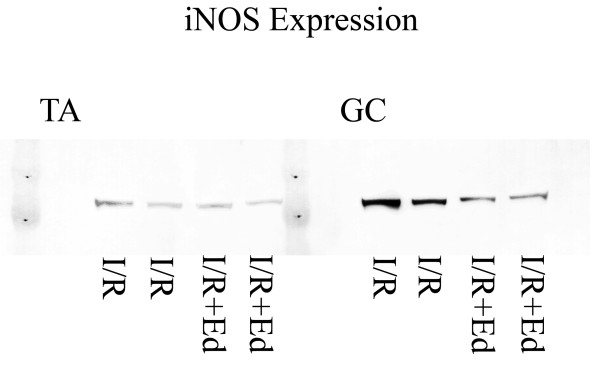
**Immunoblotting with anti-iNOS 24hours (1day) after reperfusion.** Pretreatment of edaravone decreased the level of iNOS expression.

The extent of lipid peroxidation was determined by MDA analysis in the I/R affected muscles. The mean MDA level in the muscle tissues of the I/R group was 1.8 nmol/mg protein in GC and 2.6 nmol/mg protein in TA whereas it was 0.9 nmol/mg protein in GC and 0.7 nmol/mg protein in TA of the I/R + Ed group. (Figure 4) There was a tendency towards lower peroxidation levels observed in the GC samples of the I/R + Ed group, but it was not statistically significant. (p = 0.08) The difference in the TA peroxidation levels however was significantly lower in the I/R + Ed group. (p<0.05) In addition, western-blotting revealed that pretreatment of edaravone decreased the level of iNOS expression. (Figure 5) This result suggests that scavenging of free radicals could inhibit the injuries caused by oxidative stress to myocytes [30].

## Discussion

I/R injuries are defined as the paradoxical exacerbation of cellular dysfunction and death following the restoration of blood flow to previously ischemic tissues [1]. Re-establishment of blood flow is essential to salvage ischemic tissues; however reperfusion itself causes further damage to the ischemic tissue, threatening function and viability of the organ. I/R injuries occur in a wide range of organs including the heart, kidney, liver, brain and intestines heart, liver, brain, kidney, colon and skeletal muscles [2-4,31]. Examples of I/R injuries of skeletal muscles in this study can arise as a consequence of a range of vascular events including thrombolytic therapy, organ transplantation, limb trauma, or aortic clamping during repair of abdominal aortic aneurysms. The resulting tissue destruction and edema can lead to further worsening of the physiology and increased microvessel constriction leading to compartment syndrome [32-34]. In addition, if a large volume of tissue falls into ischemia, subsequent revascularization and hence reperfusion of the ischemic tissue will also induce systemic effects on distant organs, leading to a multi-system organ failure called crush syndrome [35,36]. Effective therapies counteracting these complications are not yet available in clinical settings, though there have been a number of reports regarding treatment for I/R injuries of skeletal muscles [37-39].

Despite such studies devoted to examining I/R injury of skeletal muscle and its underlying mechanisms, no universally accepted model for inducing skeletal muscle I/R injury has evolved. The Crawford model was used for the tourniquet-induced hindlimb I/R injury in this study [5]. Another model put forth by McGivney, using a hemorrhoidal ligator (MHL) band has been widely used as tourniquet-induced hindlimb I/R injury model in mice [37]. The MHL model, however, has been criticized for its inability to control for nonspecific neuromuscular damage due to the crushing force of the rubber band on the underlying tissue. Additionally, the tension gradually decreases in this band. On the other hand, the model using an orthodontic rubber band (ORB) reported by Crawford appears to be superior to the MHL band model because ORBs produce complete, consistent, and reproducible ischemia in murine models of I/R using significantly less tension than the MHL band. Our histological analysis also showed that there was little difference in the degree of injury of muscle fibers, indicating that this simple model was likely to be suitable for evaluation of the effects of treatment for skeletal muscles of I/R injury.

While there have been several clinical strategies designed to reduce the effects of I/R injuries, none of them have been broadly successful in attenuating detrimental effects. These strategies include pharmacological intervention, hyperbaric oxygen, hypothermia, and pre- and post-injury conditioning [38-40]. The potential therapeutic benefits of edaravone has been previously recognized and investigated for I/R injuries such as cerebral infarctions as there have been a number of reports that oxygen free radicals are involved in the pathogenesis of I/R injury [20,22,41]. A recent Japanese multicenter randomized clinical trial has demonstrated that edaravone, administered within 72 hours after onset of ischemic stroke significantly reduces brain infarct volume and produces sustained benefits in the functional outcomes of patients compared with placebo treatment [23]. These results led to our hypothesis that edaravone, approved in Japan as a neuro-protective agent, could protect skeletal muscles from I/R injury not only in the animal model, but also in clinical situations. Furthermore, it has been reported that application of other antioxidants has therapeutic potential for I/R injury of skeletal muscle. The present study demonstrates that pretreatment of edaravone significantly protects against I/R injury of skeletal muscles in the hindlimb of mice. This study is particularly significant for showing the therapeutic benefits of a single-dose administration of edaravone 30 minutes prior to the onset of ischemia. This preischemic treatment protocol mimics the commonly seen clinical conditions of long duration tourniquet use for extremity surgery and plastic surgeries such as free muscle transfer.

Infiltration of inflammatory cells was also inhibited by scavenging free radicals with edaravone. The final common pathway of I/R injury is edema, attraction of activated leukocytes, and the formation of membrane-attack complexes, all of which lead to disruption of cell membranes resulting cell death [17]. The oxygen free radicals initiate lipid peroxidation of cell membranes, which causes cellular dysfunction and tissue necrosis. Our results showed that one dose of pretreatment edaravone efficiently reduced lipid peroxidation and neutrophil infiltration into the ischemic muscles. Therefore, the scavenging of oxygen free radicals induced immediately after ischemia-reperfusion may not only maintain tissue viability but also enable sufficient contractile function of skeletal muscle.

Furthermore, expression of iNOS, which was shown to play an important role in the pathophysiology of I/R injury in other organs as well as inflammatory cytokine, was inhibited in the skeletal muscles treated by edaravone [20,42]. The skeletal muscles were also reported to be influenced by NO generated by inducible form of NOS [43]. Barker et al. demonstrated that the area of necrosis was greater in wild-type than in iNOS-knockout mice, the difference being significant after 90 minutes of ischemia [43]. NO is formed as a metabolic product the stepwise conversion of L-arginine guanidine nitrogen atoms [44]. The presence of an unpaired electron in the NO radical makes it highly reactive, with a half-life of only a few seconds and readily capable of reacting with other species such as superoxides. The reaction is catalyzed by the NOS enzyme, of which three isoforms have been identified, each having specific localizations and functions [29]. Three NOS isoforms has been characterized, each encoded by different chromosomes. Two enzyme isoforms are constitutively expressed (endothelial eNOS and neuronal nNOS), whereas one isoform is an inducible enzyme (iNOS), initially found in macrophages. iNOS levels are markedly increased in inflammatory conditions, such as autoimmune inflammatory myopathies. The inducible isoform of NOS becomes up regulated in response to inflammatory stimuli such as endotoxins, cytokines and lipid mediators. Surges in NO production mediated by iNOS are cytotoxic and have been implicated in the inflammatory destruction of tissues such as heart, kidney, liver, brain, intestine and skeletal muscle [45]. In our experiments expression of iNOS was inhibited in the skeletal muscles treated by edaravone. Therefore, directly inhibiting lipid peroxidation of the myocyte by free radicals in skeletal muscles may reduce the secondary edema and inflammatory infiltration incidence of oxidative stress on tissues.

## Conclusion

The injury severity score in the I/R + Ed group compared to the I/R group showed a significantly a lower rate both in GC and TA samples. While edaravone may have multiple effects, our data showed that pretreatment with edaravone protected muscle tissue from damage due to ischemia-reperfusion. We speculate that pretreatment of edaravone directly inhibits lipid peroxidation of myocytes by free radicals in skeletal muscles after I/R injury and indirectly protects myocytes from secondary damage due to edema, inflammatory infiltration and transduction of oxidative stress. Further studies are warranted to confirm the novel therapeutic potential edaravone may have for I/R injuries of skeletal muscle.

## Abbreviations

I/R: Ischemia-reperfusion; GC: Gastrocnemius; TA: Tibialis anterior; DVT: Deep venous thrombosis; Ed: Edaravone; MDA: Malondialdehyde; iNOS: Inducible nitric oxide synthase; CAPE: Caffeic acid phenethyl ester; ORB: Orthodontic rubber bands; HE: Hematoxylin and eosin; RS: Roper scientific; TBA: Thiobarbituric acid; SDS: Sodium dodecyl sulfate; SDS-PAGE: Sodium dodecyl sulfate polyacrylamide gel electrophoresis; MHL: McGivney hemorrhoidal ligator; eNOS: Endothelial nitric oxide synthase; nNOS: Neuronal nitric oxide synthase.

## Competing interests

The authors declare that they have no competing interests.

## Authors’ contributions

KH and MT were responsible for the design and coordination of the data acquisition and analysis, interpretation of the data, and the writing of the manuscript. TI and TU participated in the experimental design and techniques. HS and KA performed the pathological examinations. MH participated in statistical evaluation of the data. AU and AS did final editing. All authors read and approved the final manuscript.

## Pre-publication history

The pre-publication history for this paper can be accessed here:

http://www.biomedcentral.com/1471-2474/14/113/prepub
